# The Myxobacterium *Myxococcus xanthus* Can Sense and Respond to the Quorum Signals Secreted by Potential Prey Organisms

**DOI:** 10.3389/fmicb.2017.00439

**Published:** 2017-03-14

**Authors:** Daniel G. Lloyd, David E. Whitworth

**Affiliations:** Institute of Biological, Environmental and Rural Sciences, Aberystwyth UniversityCeredigion, UK

**Keywords:** *N*-Acyl homoserine lactone, *Myxococcus xanthus*, predation, predator-prey interaction, kairomone, signaling, sporulation

## Abstract

The myxobacterium *Myxococcus xanthus* is a predatory member of the soil microfauna, able to consume bacteria (Gram-negative, Gram-positive), archaea, and fungi. Many potential prey of *M. xanthus* communicate amongst themselves using acyl homoserine lactones (AHLs) as quorum signals. *M. xanthus* cannot itself produce AHLs, but could potentially benefit by responding to exogenous AHLs produced during signaling between proximal prey. Four AHLs of different side chain length were tested and all found to delay sporulation of *M. xanthus* vegetative cells, and to stimulate germination of myxospores, increasing the proportion of predatory vegetative cells in the population. The predatory activity and expansion rates of *M. xanthus* colonies were also found to be stimulated by AHLs. Thermally inactivated AHLs had no effect on *M. xanthus* cells, and the response to AHLs depended (non-linearly) on the length of AHL side chain, suggesting that the effect of AHLs was mediated by specific signaling within *M. xanthus*, rather than being a consequence of the chemical or physical properties of AHLs. Therefore, it seems that the presence of xenic quorum signaling molecules enhances the predatory activity of *M. xanthus*. AHLs increase the proportion of the population capable of predation, and stimulate the motility and predatory activity of vegetative cells. We therefore propose that in the wild, *M. xanthus* uses AHLs as markers of nearby prey, potentially eavesdropping on the conversations between prey organisms.

## Introduction

Our current understanding of the soil microbiome, and crosstalk therein, is limited by the complexity of the potential interactions between individual denizens (Hirsch and Mauchline, 2012). The Gram-negative myxobacterium *Myxococcus xanthus* is a predatory member of the soil ecosystem with the capacity to lyse and consume a broad range of microbial species (Morgan et al., 2010). Despite the widespread distribution of myxobacteria and their suggested role as ecological regulators, there has yet to be a definitive study into their ecological impact, or the control mechanisms used (Shimkets, 1990; Dawid, 2000).

Within the soil there are numerous species competing for nutrients and space, creating complex food webs which culminate in a small number of ‘apex’ style carbon sequesterers (Lueders et al., 2006). Throughout such competitive ecosystems, individuals often seek to enhance their long term survival via mutual cooperation, which imposes a fitness cost on the individual, but increases the fitness of the population as a whole (Strassmann et al., 2011). The formation of a biofilm structure is the most commonly characterized form of bacterial cooperation, providing communal resistance to various environmental stresses (Watnick and Kolter, 2000; Jefferson, 2004). In order to orchestrate population-wide behaviors such as biofilm formation, quorum signaling (QS) systems are often used by bacteria to regulate gene expression (Miller and Bassler, 2001). There have been numerous systems characterized, each producing and detecting highly specific quorum signals (Dong and Zhang, 2005; Waters and Bassler, 2005).

A typical QS system involves members of a population producing a specific signaling molecule, which diffuses out of the producing cell and accumulates in the local environment. The concentration of QS molecule is thus broadly indicative of the number of signal-producing cells. At a particular threshold concentration of signal (representing a quorum of producing organisms), a biological response to the signal is triggered, typically through collective regulated changes in gene expression (Bassler and Losick, 2006).

Gram-negative bacteria primarily use acyl homoserine lactones (AHLs) as their quorum signals, whereas Gram-positive species often use processed oligopeptides for communication. All AHLs contain the same core structure containing a lactone ring, with a variable R-group carbon chain which can differ in chain length and with the addition of side chains (Miller and Bassler, 2001). AHLs are made by LuxI homologs (AHL synthases), are sensed by LuxR homologs, and are rapidly degraded in the environment through ring-opening lactonolysis (Yates et al., 2002). On binding AHL, LuxR binds to specific operator sites, activating expression of target genes, including but not limited to *luxI* and *luxR* (Williams et al., 2007). The resulting positive loop has led to QS molecules commonly being described as autoinducers.

Myxobacteria are renowned for their multicellular lifecycle, which features group motility, social development, and co-operative predation, however, their social interactions are not mediated by AHLs (Muñoz-Dorado et al., 2016).

The ability to control cellular motility enables *M. xanthus* to actively seek new nutrient sources and invade prey colonies (Berleman and Kirby, 2009). Motility of *M. xanthus* is encoded by two distinct genetic systems. A-motility (adventurous) can be performed by individual cells, while S-motility (social) requires cells to work in concert (Hodgkin and Kaiser, 1979). Upon starvation, populations of cells orchestrate their motility, forming a series of distinct patterns, culminating in the formation of a fruiting body containing 10^5^ cells (Zusman et al., 2007; Kaiser et al., 2010; Muñoz-Dorado et al., 2016).

The developmental program which results in fruiting body formation involves three chemically characterized intercellular signals. The A-signal is a diffusible population density-dependent signal which acts as a quorum signal. A-signal is not, however, an AHL, but rather a mixture of proteases, peptides, and amino acids (Kaplan and Plamann, 1996). C-signal is a surface-associated signal which is exchanged between cells upon cell–cell contact, while the E-signal is a mixture of fatty acid iso-15:0 and diacylmonoalkyl ether lipid TG1 (Ahrendt et al., 2016). A-signal acts as a signal of starvation and population density, initiating the developmental program. As starvation progresses, C-signaling induces its own production, leading to progressively higher signaling on cell–cell contact. As levels of C-signal increase, cells progressively ripple, stream, aggregate, and then sporulate within the nascent fruiting body (Kim and Kaiser, 1990; Muñoz-Dorado et al., 2016).

When myxospores are returned to a nutrient rich environment they germinate, reverting to predatory vegetative cells (Otani et al., 1995). The conversion from myxospore to vegetative cell comprises at least two distinct steps; a loss of refractility and spore coatings, followed by an increase in cellular metabolism and the emergence of a rod shaped cell (Elías and Murillo, 1991). Collective germination of the spores within a fruit results in a population of germinants, immediately proficient at co-operative predation.

*Myxococcus xanthus* colonies use the co-operative release of extracellular digestion factors and secondary metabolites to enable predation upon a broad range of prey (Rosenberg et al., 1977; Cao et al., 2015). Protease secretion and growth rate are cell density-dependent (Rosenberg et al., 1977), implying co-operative action, and secreted public goods include outer membrane vesicles (OMVs) which can kill and lyse prey organisms (Evans et al., 2012).

Although *M. xanthus* does not make AHLs itself, we would nevertheless expect it to be surrounded by the AHLs produced by other soil (potentially prey) bacteria. We therefore tested whether QS molecules made by (and indicating the presence of nutrients in the form of) typical soil bacteria, might affect the nutrient-dependent social behaviors of myxobacteria.

## Results

### AHLs Enhance *M. xanthus* Motility

**FIGURE 1:**
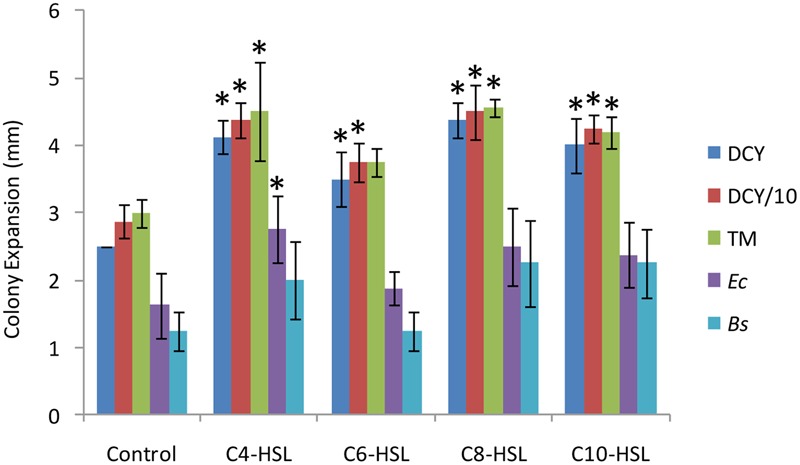
**Motility rates in response to nutrients, acyl homoserine lactones (AHLs) and lawns of prey.** Colony expansion was measured (in mm) for quadruplicated colonies 48 h after spotting. Colonies were plated onto DCY (rich medium), reduced nutrient medium (DCY/10), TM (nutrient free medium), a lawn of *Escherichia coli* (*Ec*) on TM, and a lawn of *Bacillus megaterium* (*Bs*) on TM. The response to four AHLs was measured on DCY/10 medium supplemented at a concentration of 6 μM. Error bars represent ± 1 standard deviation, and asterisks indicate a statistically significant difference from the control (*P* < 0.05).

To test for any effect of QS molecules on motility, colony expansion assays were performed in the presence and absence of four AHLs (C4-HSL, C6-HSL, C8-HSL, and C10-HSL). As can be seen in **Figure 1**, the presence of AHLs significantly increased the distance swarmed after 48 h on DCY, DCY/10, and TM media (*P* < 0.05). The rate of swarm expansion was not significantly affected by the nutritional value of the medium (DCY, DCY/10 or TM), but the size of the stimulatory effect depended on the specific AHL being tested. C6-HSL had the least effect on motility, but it still significantly increased swarming over the control on DCY and DCY/10, although not on TM. There was no significant difference in motility comparing between the addition of C4-HSL, C8-HSL, and C10-HSL, with all three stimulating colony expansion by approximately 50%.

**FIGURE 2:**
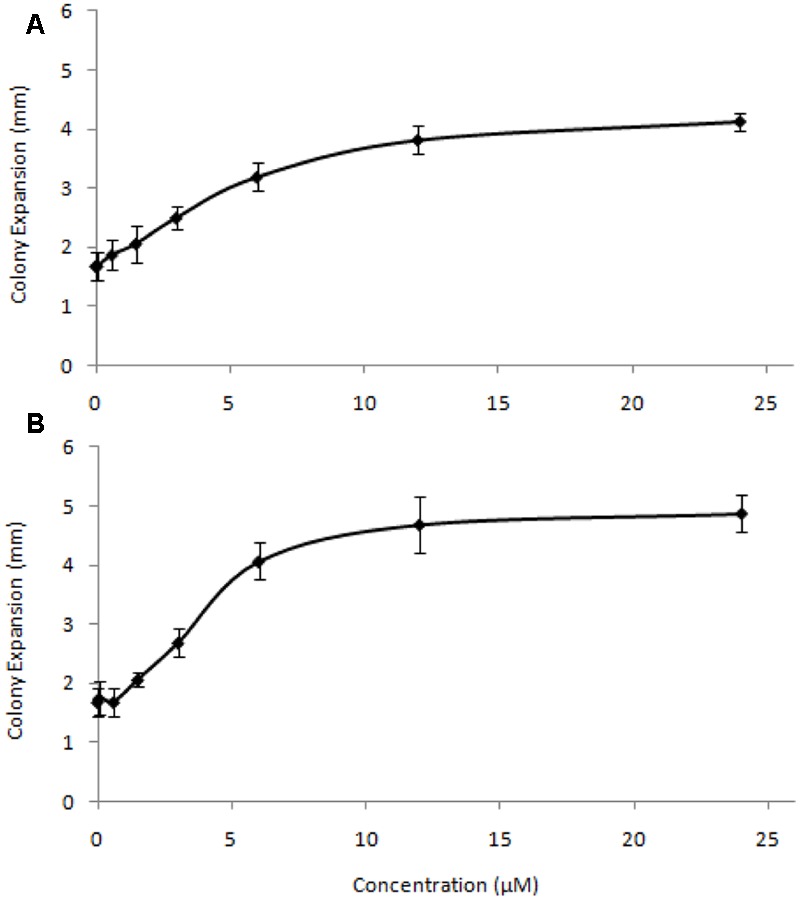
**Concentration-response relationship for C4-HSL (A)** and C10-HSL **(B)**. Colony expansion was measured (in mm) for quadruplicated colonies on DCY/10 at a range of AHL concentrations. Error bars represent ± 1 standard deviation.

To test the effective concentrations of C4-HSL and C10-HSL, colony expansion rates were assessed across a range of AHL concentrations (**Figure 2**). For both AHLs tested the enhancement of motility saturated at concentrations above 10μM, with half-maximal effects seen around 1.5–2 μM. The response to C4-HSL was hyperbolic, while at low concentrations of C10-HSL the response appears sigmoidal, potentially suggesting a threshold minimal effective concentration. Heat-inactivated AHLs (100°C for 120 min) exhibited no stimulation of motility at any concentration.

### AHLs Retard Developmental Sporulation and Promote Myxospore Germination

**FIGURE 3:**
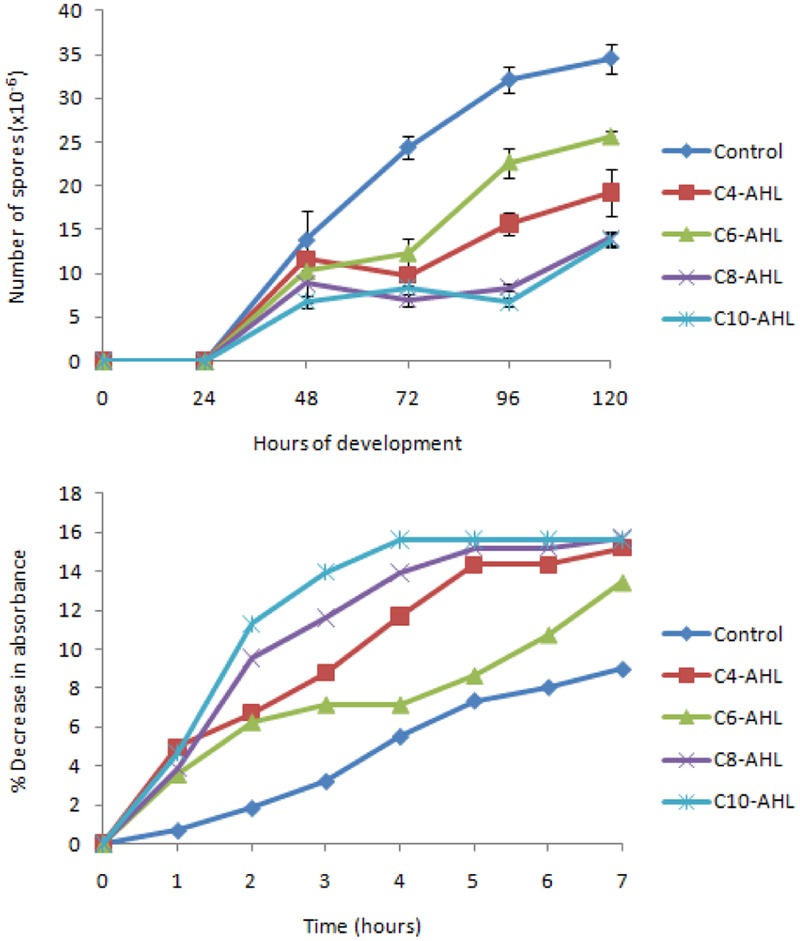
**The balance between spores and vegetative cells is affected by AHLs.** Sporulation **(A)** of cells was measured in quadruplicate (error bars denote 1 standard deviation). All AHLs gave significantly fewer spores than the control at 72, 96, and 120 h (*P* < 0.05). Germination **(B)** was indicated by a loss of refractility (in triplicate), and here the mean is expressed as a % decrease from the mean absorbance at time = 0.

The effect of AHLs on the production of fruiting body myxospores was also assayed. At time = 0 vegetative cells were spotted onto nutrient free medium and the number of heat and sonication resistant spores formed measured as a function of time (**Figure 3A**). From time = 72 h onward, each AHL tested was found to retard the formation of spores, with significantly fewer spores than the control (*P* < 0.05). Again, individual AHLs gave rise to different magnitudes of effect, with C8-HSL and C10-HSL exhibiting the greatest effects (with only one third the number of spores produced by the control after 120 h), and C6-HSL having the least (but still significant) effect after 120 h.

The loss of refractility associated with the germination of spores was followed in the presence and absence of AHLs. The addition of AHLs increased the rate with which refractility was lost compared to control spores (**Figure 3B**). Again C6-HSL had the smallest effect, while the largest effects were produced by C8-HSL and C10-HSL. In the presence of C10-HSL loss of refractility was complete after 4 h, however, in the absence of AHLs, the refractility of control spores continued to fall after 7 h.

### Predation Is Stimulated by AHLs

One way of quantifying predatory activity is to spot predators onto a lawn of prey and measure the diameter of the zone of prey killing as a function of time. We performed such assays using both Gram-negative and -positive prey (*Bacillus megaterium* and *Escherichia coli*, respectively) in the presence and absence of AHLs. All AHLs except C6-HSL increased the size of the zone of prey killing (**Figure 1**), with C4-HSL having a significant effect (*P* < 0.05) on *E. coli* predation.

Another way of assessing predatory activity involves incubating prey cells with samples of predator and assessing % survivors (Evans et al., 2012). Using this assay to quantify predatory activity showed a significant (*P* < 0.005) stimulatory effect of AHLs on killing by cells of *M. xanthus*. Under conditions in which *M. xanthus* cells killed 47 ± 5% (mean ± 1 standard deviation) of prey cells, the addition of AHLs to the assay gave 75 ± 6% killing. There was no significant difference between the effects of the four different AHLs.

In order to define which aspects of predatory activity are enhanced by AHLs, we tested whether AHLs added exogenously increased the killing activity of OMVs, whether incubation of producing cells with AHLs resulted in them producing more active OMVs, and whether incubation with AHLs increased the secretion of OMVs. Incubation of OMV-producing cells with AHLs neither increased the amount of OMVs produced, nor did it produce OMVs (or supernatant) with increased predatory activity. Addition of AHLs to OMV preparations did not stimulate their predatory activity either, suggesting that the AHL-mediated stimulation of predatory activity is not mediated mechanistically through OMVs.

## Discussion

The addition of AHLs affects several behaviors exhibited by *M. xanthus*. Several lines of evidence suggest this is a consequence of signaling rather than due to the chemical and/or physical properties of the added AHLs. Motility, as manifested by colony expansion rates, was insensitive to nutrient levels in the substrate (**Figure 1**), but was stimulated by AHLs at very low concentrations (2 μM gave half-maximal stimulation), implying that AHLs are not being used as a nutrient source. In addition, the magnitude of the behavioral response in *M. xanthus* differed depending on the specific AHL added. This variation is non-linear with respect to AHL side chain length, showing that *M. xanthus* has differing sensitivity toward specific AHLs. Loss of activity upon heating [presumably due to enhanced pH-dependent ring-opening lactonolysis (Yates et al., 2002)], also indicates an effect due to specific signaling rather than non-biological properties.

The addition of AHLs shifted the balance between vegetative cells and myxospores toward predatory vegetative cells. In addition, the predatory activity of vegetative cells was increased by AHLs. It is possible that the increase in predatory activity is purely a consequence of augmented motility rates increasing contact between prey and predator. This is plausible, given the antimicrobial activity of OMVs was unaltered in any way by AHLs, although we only tested activity against *E. coli* prey, and potentially AHLs could enhance the activity of OMVs against other prey organisms. Regardless of mechanism, AHLs indicative of prey stimulate predatory activity, and increase the predatory proportion of the population.

It is already known that *M. xanthus* is able to sense and respond to the presence of prey. The lipid phosphatidyl ethanolamine (PE) is a chemoattractant and its release from lysed prey potentially recruits further predators to the vicinity (Bonner and Shimkets, 2006). Following the invasion of a prey colony *M. xanthus* uses a chemosensory pathway to direct colony movement in a manner that allows for complete lysis of prey (McBride and Zusman, 1996; Berleman et al., 2006), and there is also the potential for prey to attract *M. xanthus* through elasticotaxis (Fontes and Kaiser, 1999).

It is thought that PE makes a suitable chemoattractant for *M. xanthus* because of the myxobacterium’s slow gliding speed. The low diffusion rate of PE in aqueous media makes a short range but stable concentration gradient to which *M. xanthus* can slowly respond (Bonner and Shimkets, 2006). AHLs are short lived due to pH-dependent lactonolysis (Tan et al., 2015), which means that long range signaling is precluded, and AHL gradients can only be established if there is continued production, i.e., by high densities of living prey (Nilsson et al., 2001).

The sensation of a signal by an organism other than the intended recipient means that the response of *M. xanthus* to AHLs could potentially represent a form of microbial eavesdropping, and AHLs as kairomones. Amongst metazoans, eavesdropping on chemical signals by predators has only been documented relatively recently (Hughes et al., 2010) compared to a wealth of literature regarding eavesdropping on acoustic signaling (Strauß and Stumpner, 2015). Bacterial eavesdropping on quorum signals has been described previously, with antagonistic and synergistic consequences (Chandler et al., 2012; Michie et al., 2016; Papenfort and Bassler, 2016), however, the work presented here seems to be the first example of a bacterial predator responding to signals produced by prey microbes. AHLs that stimulatex predation are produced by many known *M. xanthus* prey organisms, including *Serratia marcescens, Pseudomonas syringae* (Pham et al., 2005), *P. aeruginosa* (Evans et al., 2012), and *Rhizobium sp.* (our unpublished data), and we would therefore expect predatory eavesdropping to be widespread in soils. AHLs have been detected in biofilms at concentrations in excess of 600 μM, (Charlton et al., 2000), so the concentrations used in this study (up to 25 μM, with half-maximal effects at 2 μM) are likely to be encountered by *M. xanthus* in the wild.

It is not clear mechanistically how *M. xanthus* could respond to AHLs. No myxobacterial genome sequence encodes a LuxI AHL synthase (Pfam domain PF00765), and only one myxobacterium (*Haliangium ochraceum*) encodes a full-length LuxR homolog (Hoch_3098) containing the N-terminal autoinducer binding domain (PF03472). The P2TF database of DNA-binding proteins lists 12 proteins encoded in the *M. xanthus* genome which contain the C-terminal LuxR DNA-binding domain (PF00196). Six of those contain no recognizable sensory domain while five have N-terminal two-component system receiver domains, including the regulator of fruiting body development FruA.

The suppression of sporulation by AHLs does, however, open possibilities for identification of the genes responsible. Following random mutagenesis, enrichment of mutants with disrupted AHL-sensitivity could be achieved using individual-colony screens (Yu et al., 2016) in the presence of AHLs, followed by heat-killing of vegetative cells. It would also be interesting to determine which *M. xanthus* genes are differentially regulated on exposure to AHLs by using transcriptome sequencing methods. This would provide insights into whether enhanced predation is just a consequence of increased motility and an imbalance of vegetative cells:myxospores, or whether expression of ‘predatory’ genes is actively induced.

If the hypothesis of predatory eavesdropping by *M. xanthus* is correct, we would expect to see increased fitness of *M. xanthus* when predating an AHL-producing prey compared to a non-AHL producing prey. Experiments investigating such a phenomenon will be non-trivial to undertake though, as AHLs affect the biology of the producing organism, usually making them more resistant to environmental stressors (and presumably more recalcitrant to predation). It would also be difficult to disentangle AHL-induced increases in predatory activity from the alternative interpretation of AHL-enhanced susceptibility of prey to predation. We would also predict that other types of QS signaling molecules commonly secreted by prey (including peptides, farnesol, AI-2, A-factor, etc.) might affect myxobacterial predatory behavior, and that the addition of xenic signals might stimulate directional swarming toward the signal source.

Our understanding of trophic levels and predator-prey relationships within polymicrobial communities is currently limited, but receiving increasing research attention. The demonstration of a predator responding to signals secreted by prey, suggests that predator-prey interactions can be much more sophisticated than mere chance encounters between predator and prey, and phenomena such as QS, which exhibits a clear fitness advantage in the laboratory, may well have profound downsides in the wild.

## Materials and Methods

### Bacterial Strains, Media, and Cultivation

*Myxococcus xanthus* wild-type strain DK1622 (Kaiser, 1979) was used throughout, maintained on the rich medium DCY as described previously (Whitworth et al., 2008), with incubation at 30°C. Low nutrient DCY/10 medium is DCY diluted to 10% v/v. Media were solidified with the addition of 1.5% agar. TM was used as a nutrient free medium for developmental assays (Whitworth et al., 2008). *E. coli* strain TOP10 (Invitrogen) and *B. megaterium* (strain NCIMB 4821) were cultivated on LB (Sambrook et al., 1989), solidified with 1.5% agar as required. Cells were harvested from liquid cultures by centrifugation (4000 × *g* for 10 min). OMVs and soluble culture supernatant were purified from late-exponential cultures of *M. xanthus* by differential centrifugation as described previously (Evans et al., 2012).

### Signaling Compounds

*N*-Acyl homoserine lactones (5 mg of >95% purity determined by high field proton NMR spectroscopy) were acquired from the University of Nottingham, dissolved in ethylacetate and aliquots lyophilised overnight before storage at 5°C. AHLs were dissolved in TM to a stock concentration of 2 mM immediately before being used at a working concentration of 6 μM. Control experiments used TM added to a lyophilised aliquot of ethylacetate without AHL. Heat-inactivation of AHLs was performed in a liquid state, on aliquots that had been resuspended in TM.

### Physiological Assays

To monitor developmental sporulation, cells from late-exponential phase cultures were sedimented, washed with TM and spotted onto TM plates. Harvested spots were scraped from plates and spores enumerated as described previously (Higgs and Merlie, 2007). To assess motility rate, concentrated cells were spotted onto solid media as described by Higgs and Merlie (2007) and the increase in colony size recorded. To mark the original extent of the colony, Indian Ink was added to the cell suspension to a concentration of 0.1% v/v prior to spotting. To assess germination, spores were produced as before, enumerated, and added to DCY liquid medium and incubated at 30°C. As spores germinate they lose refractility, which was monitored as a decrease in turbidity at 560 nm as described by Otani et al. (1995). Killing of *E. coli* by OMVs and culture supernatant was assessed by killing plate assays as described by Evans et al. (2012), in which a known number of *E. coli* cells are mixed and incubated with a sample, and then plated to enumerate viable survivors.

The statistical significance of differences between swarm expansion rates, numbers of spores, and *in vitro* killing activities were assessed by one-way ANOVA (*P* < 0.05), with *post hoc* Tukey tests.

## Author Contributions

DL designed and performed the experiments. DW conceived and supervised the project. DL and DW analyzed the data, wrote and edited the manuscript.

## Conflict of Interest Statement

The authors declare that the research was conducted in the absence of any commercial or financial relationships that could be construed as a potential conflict of interest.
